# Evaluation of iron stores in hemodialysis patients on maintenance ferric Carboxymaltose dosing

**DOI:** 10.1186/s12882-019-1263-8

**Published:** 2019-03-01

**Authors:** Matthias Diebold, Andreas D. Kistler

**Affiliations:** Division of Nephrology, Department of Medicine, Cantonal Hospital Frauenfeld, Pfaffenholzstrasse 4, 8501 Frauenfeld, Switzerland

**Keywords:** Anemia of chronic kidney disease, Iron, Ferritin, Transferrin saturation, Chronic hemodialysis, Ferric carboxymaltose, FCM

## Abstract

**Background:**

Iron is administered intravenously (IV) to many dialysis patients at regular intervals and iron stores are evaluated through periodic measurements of ferritin and transferrin saturation (TSAT). In patients without kidney diseases, large single doses of IV iron lead to a transient rise in serum ferritin that does not reflect iron stores. It is not known whether and to what extent smaller IV iron doses used to maintain adequate stores in hemodialysis patients lead to transient spurious elevations of ferritin and TSAT.

**Methods:**

Ferritin and TSAT were serially determined over four weeks after the administration of ferric carboxymaltose (FCM) in hemodialysis patients on a stable maintenance FCM dosing regimen of 100 mg or 200 mg every four weeks.

**Results:**

Ferritin values increased by 113 ± 72.2 μg/l (*P* < 0.001) from baseline to the peak value and remained significantly elevated until two weeks after the administration of 100 mg FCM (*n* = 19). After the administration of 200 mg FCM (*n* = 12), ferritin values increased by 188.5 ± 67.56 μg/l (*P* < 0.001) and remained significantly elevated by the end of week three. TSAT values increased by 12.0 ± 9.7% (*P* < 0.001) and 23.1 ± 20.4% (*P* = 0.002) in patients receiving 100 or 200 mg FCM, respectively, and returned to baseline within four days.

**Conclusions:**

IV administration of FCM at doses of 100 or 200 mg in hemodialysis patients leads to dose-dependent transient ferritin elevations of extended duration. Temporal coordination of blood sampling for iron status evaluation with the maintenance IV iron dosing schedule is advisable.

**Trial registration:**

ISRCTN12825165 (retrospectively registered 01/02/2019).

**Electronic supplementary material:**

The online version of this article (10.1186/s12882-019-1263-8) contains supplementary material, which is available to authorized users.

## Background

Anemia is nearly universal among patients with advanced chronic kidney disease (CKD), contributes to morbidity and affects quality of life in dialysis patients [1]. The etiology of anemia in CKD is multifactorial and driven by both, reduced production and decreased survival of red blood cells (RBC). Erythropoietin stimulating agents (ESAs) have revolutionized the treatment of renal anemia but are costly and potentially harmful [2–5], particularly if used at high doses [6]. These observations have shifted the focus of anemia treatment in CKD towards strategies beyond the application of ESAs, particularly restoration of adequate iron stores.

Chronic hemodialysis patients are prone to negative iron balance due to reduced dietary intake, impaired absorption, and blood loss during dialysis, regular blood sampling as well as occult intestinal blood loss. Two strategies for IV iron administration in hemodialysis (HD) patients may be used, periodic or maintenance dosing [7, 8]. Periodic iron repletion consists of a series of iron doses administered over a short period of time aiming to replenish iron stores, followed by an interval without iron administration. Maintenance dosing consists of smaller doses given in regular intervals aiming to maintain stable iron stores.

Several intravenous iron formulations are available and licensed for use in hemodialysis patients [9]. Ferric carboxymaltose (FCM) is a relatively new formulation with a complex carbohydrate shell that tightly binds elemental iron, allowing a large dose to be administered in a relatively short period of time. In dialysis patients, FCM is labelled for the administration of up to 200 mg per dialysis session. Common maintenance dosing schedules of FCM consist of 100 or 200 mg given every 2–4 weeks.

Ferritin and transferrin saturation (TSAT) are used to assess iron status in dialysis patients. Although no evidence-based targets exist [10] and recommendations differ to some extent [7, 11–14], adjustments of the dose and frequency of IV iron administration are usually based on these periodically measured laboratory values. The 2012 KDIGO guidelines on anemia management [7] recommend evaluating iron stores by measurement of ferritin and TSAT at least every three months. Notably, neither the KDIGO guidelines nor other recommendations specify the optimal timing of iron status evaluation relative to the last IV iron administration in HD patients. In subjects without CKD, a single infusion of sodium ferric gluconate [15] or FCM [16] leads to a transient rise of ferritin, which does not reflect iron stores but rather the iron-induced secretion of ferritin by hepatocytes [17]. Therefore, we hypothesized that IV iron administrations in hemodialysis patients might lead to similar transient changes in ferritin values. Iron status evaluation is performed on a regular schedule in most dialysis centers (usually in three-month intervals) and may not be coordinated with the timing of iron administration if maintenance iron dosing is applied. Thus, iron parameters might be influenced by the timing of their determination relative to the last IV iron administration. The aim of this study was to evaluate the dynamic change of laboratory iron parameters in patients on a stable four-weekly maintenance regimen of FCM at the labelled and commonly used doses of 100 or 200 mg in hemodialysis patients.

## Methods

### Study design

We conducted a prospective observational study in two dialysis units run by the same hospital-based nephrology division (the cantonal hospital of Frauenfeld, Switzerland). The study was initiated in June 2017, the last patient visit was in February 2018. The observation period for every patient started with a scheduled administration of FCM and ended with the next scheduled dose of FCM after four weeks. Neither the FCM dosing schedule nor any other drug exposure was influenced by the study investigators. The study was approved by the Ethics committee of Eastern Switzerland (Ethikkommission Ostschweiz, EKOS) and conducted in adherence to the Declaration of Helsinki. All patients gave written informed consent.

### Study population

Chronic hemodialysis patients receiving either 100 or 200 mg FCM every four weeks were eligible. Further eligibility criteria were: age 18 years or older; HD treatment for at least three months; thrice weekly hemodialysis; a stable FCM dosing schedule for the last two months or longer; a stable ESA dose for patients receiving ESAs (defined by dose adjustments of < 25% within the last two months); hemoglobin values between 95 g/l and 125 g/l within the last 12 weeks with a difference between the lowest and the highest value of < 15 g/l. Exclusion criteria were: clinical evidence of significant blood loss within the last 12 weeks (e.g. gastrointestinal bleeding); significant inflammation (CRP > 15 mg/l); hospital admission within the last month; or significant bacterial infection (e.g. pneumonia) within the last 12 weeks.

### Study assessments

**Fig. 1:**
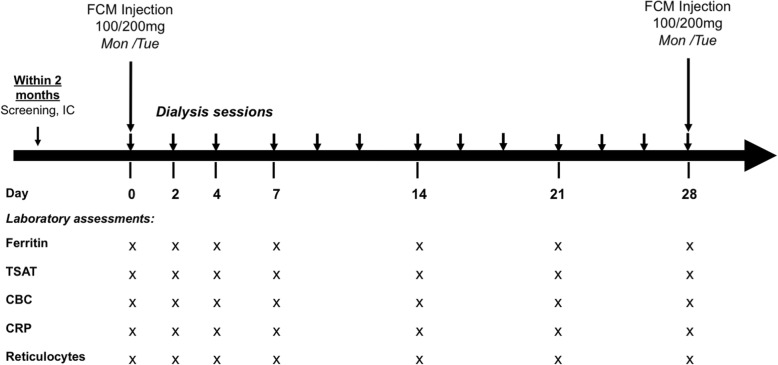
Timeline of the laboratory assessments. Abbreviations: IC, informed consent; FCM, ferrum carboxymaltose; TSAT, transferrin saturation; CBC, complete blood count; CRP, C-reactive protein

Demographic information and medical history were collected within two months before the study baseline visit. The baseline visit was defined by the administration of a 100 or 200 mg FCM bolus. FCM was administered during the last 15 min of the first dialysis session of the week (Monday or Tuesday). Blood for laboratory analyses was drawn at the beginning of the dialysis sessions at baseline, at every dialysis session in the first study week and weekly thereafter until the next FCM dose. We assessed ferritin, transferrin, free iron, complete blood count and reticulocytes, CRP, phosphate and potassium with every blood sampling. The study schedule is graphically depicted in Fig. 1. All blood samples were analyzed immediately.

### Study outcomes

The coprimary outcomes were change in serum ferritin and TSAT from baseline to the peak value, assessed separately for the 100 mg and the 200 mg FCM study arms. Secondary outcomes were time to normalization of these values (i.e. time when the mean values were no longer different from baseline or when ferritin and TSAT values were < 100 μg/l and < 10% above baseline, respectively, in 75% of patients) as well as change of ferritin and TSAT for every time point assessed. Further secondary endpoints were changes in other laboratory parameters assessed.

### Statistical analyses

Due to the lack of previous data on the expected change in ferritin and TSAT and its standard deviation, we therefore based the sample size calculation on the following assumptions: we defined 100 μg/l and 10% as the minimum change in serum ferritin and TSAT, respectively, that we considered clinically relevant. Assuming a standard deviation of 125 μg/l and 12.5% for the change in these parameters, respectively, a sample size of 12 would be required to detect a clinically relevant change with 80% power using a two-sided paired t-test with an alpha level of 0.05. Expecting a drop out of 20%, we aimed to include at least 15 patients in each study arm.

Statistical analyses were performed using SPSS version 22.0 (IBM Corporation, Armonk, NY). We used the absolute rather than the relative change in serum ferritin as outcome variable, because the former but not the latter was independent of ferritin at baseline (Additional file 1: Figures S1 and S2). A two-sided paired t-test was used to compare all serum ferritin and TSAT measurements to their baseline values after confirming their normal distribution using a Kolmogorow-Smirnow test.

In patients who developed a significant infection requiring hospital admission or antibiotic treatment during the study period, all measurements obtained thereafter were excluded from the primary analysis. In patients who developed a minor infection (e.g. viral upper respiratory tract infection), ferritin and TSAT values were exclude from analysis until one week after normalization of CRP (*<* 20 mg/l).

We performed a sensitivity analysis repeating all calculations after excluding patients with missing values and patients with a change in ferritin or TSAT from baseline to day 28 that exceeded the SD of the change of the respective values from baseline to the maximum value in the respective dosage arm.

## Results

### Patients and demographics

**Fig. 2:**
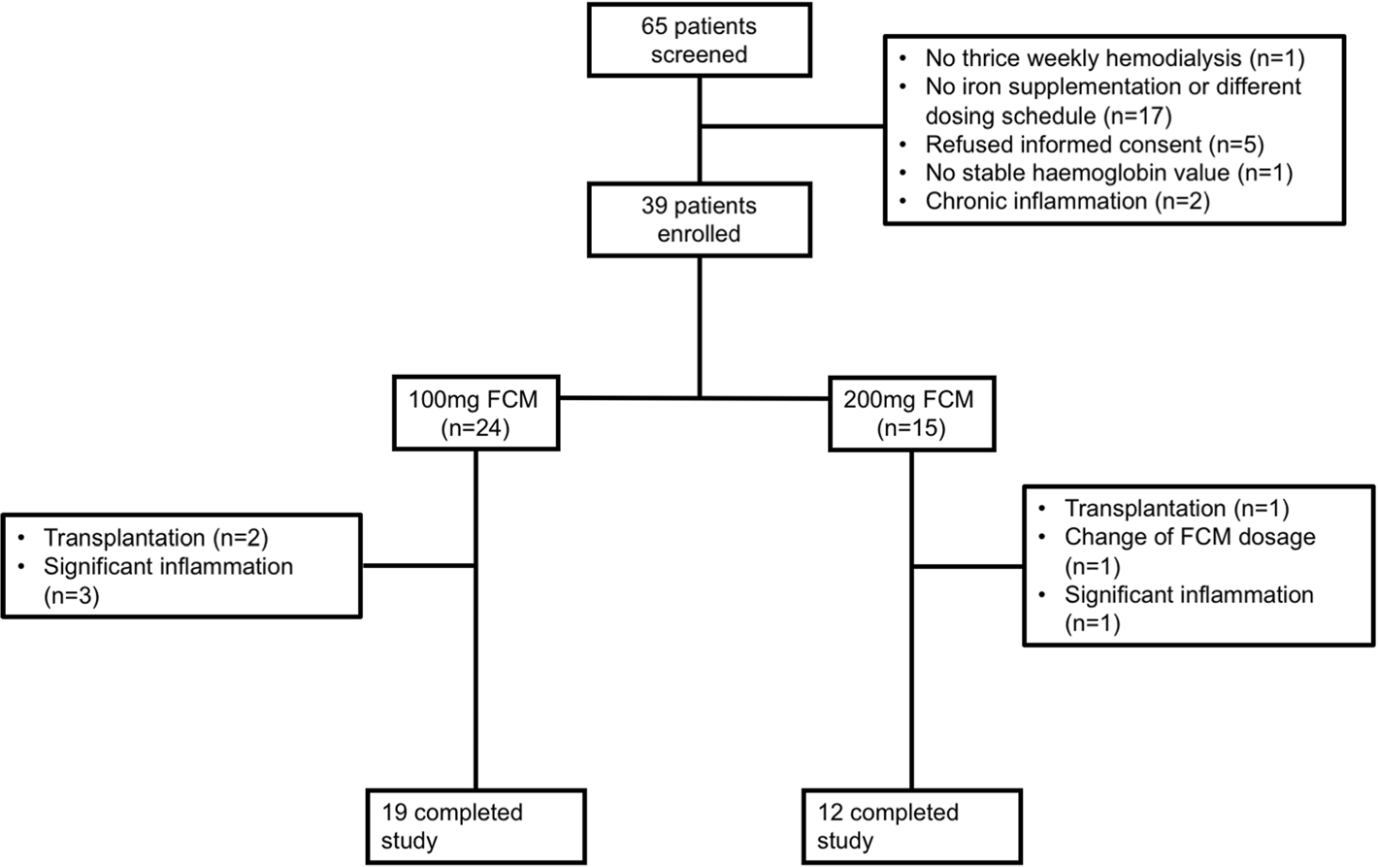
Study flowchart. Drop outs included three patients that were transplanted before the baseline visit; four patients experiencing an infection that led to a significant rise of CRP (one before the baseline visit, three on day 2) and one patient, in whom the FCM dose was changed after obtaining study consent but before the first study measurement. Abbreviations: FCM, ferrum carboxymaltose

Among 65 hemodialysis patients who were screened for the study, 39 patients were eligible and gave written informed consent (24 receiving 100 mg and 15 receiving 200 mg FCM every four weeks). Of these, eight patients were excluded from the primary analysis due to early drop out (before or directly after the baseline measurement), leaving 31 patients for the primary analysis (19 receiving 100 mg and 12 receiving 200 mg FCM every four weeks). The reasons for non-eligibility and for drop out are depicted in the study flow chart (Fig. 2). Baseline demographic and clinical characteristics of all patients are summarized in Table 1. There were no statistically significant differences in these baseline parameters between patients receiving 100 mg and those receiving 200 mg FCM, although the group sizes were small for such a comparison.

**Table 1:** Baseline characteristics of study participants

	All	100 mg FCM	200 mg FCM	*p*-value*
		*n* = 19	*n* = 12	
Age, years (range)	63.9 (34–84)	66.2 (46–84)	60.25 (34–80)	0.2
Male gender, *n* (%)	17 (54.8)	10 (52.6)	7 (58.3)	0.8
BMI, kg/m^2^	27.5 (7.4)	27.3 (8.6)	27.9 (5.4)	0.8
Dialysis Vintage, month	41.7 (34.3)	50 (38.1)	28.4 (22.9)	0.5
Erythropoiesis stimulating agents *n* (%)	28 (90.3)	16 (84.2)	12 (100)	
Dose darbepoetin alfa, μg/week	25.9 (14.7)	28.6 (13.8)	22.3 (15)	0.27
Baseline laboratory parameters				
Ferritin, μg/l, median (IQR)	425 (291–548)	450 (390–573)	313 (193–538)	0.3
TSAT, %, median (IQR)	27 (21.8–30.8)	26.1 (20.7–28.4)	29.0 (23.1–31.8)	0.3
Hemoglobin, g/l	109.5 (8.9)	107.5 (8.2)	112.8 (9.3)	0.1
CRP, mg/l, median (IQR)	2 (2–5)	2 (2–7)	3 (1.25–4)	0.7
PTH, pmol/l^1^, median (IQR)	30.4 (18.6–44.5)	32.2 (11.5–44.5)	27.5 (20.3–55.6)	0.5
Vascular access, *n* (%)				
AV-Fistula	24 (77.4)	13 (68.4)	11 (91.7)	
AV-Graft	3 (9.7)	3 (15.8)	0 (0)	
Catheter	4 (12.9)	3 (15.8)	1 (8.3)	
Primary cause of ESRD				
Diabetes	8 (25.8)	3 (15.8)	4 (33.3)	
Hypertension	3 (9.7)	2 (10.5)	1 (8.3)	
PKD	1 (3.2)	1 (5.3)	0	
GN	5 (16.1)	5 (26.3)	0	
Interstitial KD	3 (9.7)	3 (15.8)	0	
CAKUT	4 (12.9)	1 (5.3)	2 (16.7)	
Other/unknown	7 (22.6)	4 (21.1)	5 (41.7)	
Comorbidity, n (%)				
History of Cancer	11 (35.5)	8 (42.1)	3 (25)	
Chronic heart failure	8 (25.8)	6 (31.6)	2 (16.7)	
Peripheral artery disease	9 (29.0)	7 (36.8)	2 (16.7)	
Diabetes, *n* (%)	13 (41.9)	9 (47.4)	4 (33.3)	

Values are mean (standard deviation) if not otherwise stated

*TSAT* transferrin saturation, *CRP*. C-reactive protein, *PTH* parathyroid hormone; *PKD* polycystic kidney disease, *GN* glomerulonephritis, *Interstitial KD* Interstitial kidney disease, *CAKUT* congenital anomalies of the kidney and urinary tract, * *p*-values are given for the comparison between the 100 mg and the 200 mg FCM groups using T-test, Chi-square test or Mann-Whitney U-test as appropriate. ^1^ to convert PTH values from pmol/l to pg/ml, multiply with 9.43

### Iron parameters

#### Ferritin

**Fig. 3:**
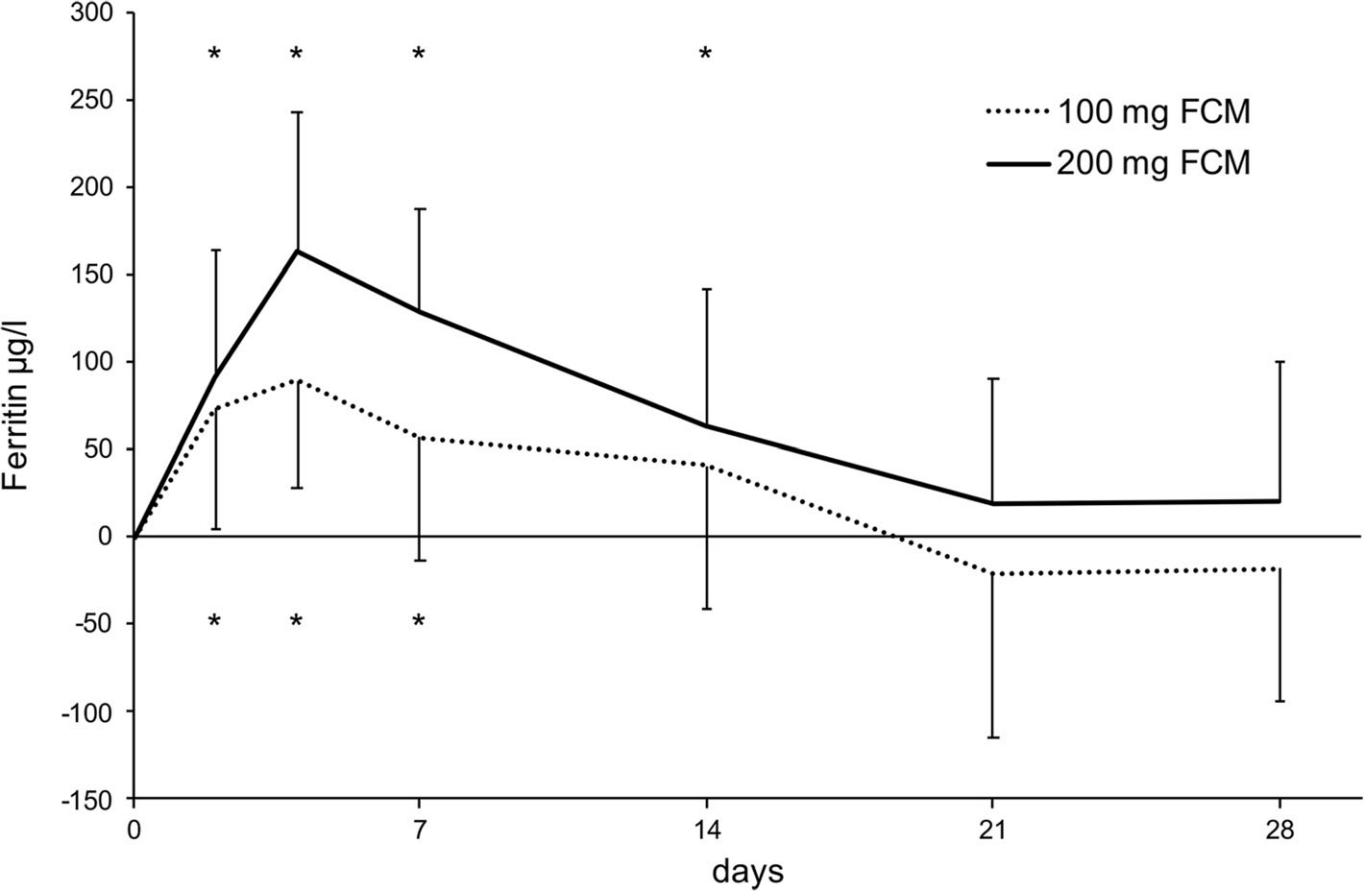
Mean change of ferritin from baseline values during 28 days after infusion of 100 mg or 200 mg FCM. Asterisks indicate a significant difference of ferritin values compared to their baseline values using a paired T-test

The change from baseline to the peak value of ferritin in relation to the corresponding baseline value for every individual patient is depicted in Additional file 1: Figure S1. In patients receiving 100 mg FCM, the mean rise in ferritin from baseline to the peak value was 113 ± 72.2 μg/l (*P* < 0.001). The peak value was observed on day 2 in nine patients, on day 4 in thirteen patients, on day 7 in seven patients and on day 14 in two patients. A significant difference of ferritin values from baseline was observed on day 2 (73.5 ± 68.7 μg/l, P < 0.001), day 4 (89.5 ± 60.9 μg/l, P < 0.001) and day 7 (56.7 ± 70.3 μg/l, *P* = 0.003) (Fig. 3). 10/19 patients (52.6%) showed a rise in ferritin from baseline to peak of ≥100 μg/l. Ferritin levels returned to < 100 μg/l above baseline in 14/19 patients (73.7%) patients by day 7, in 11/15 patients (73.3%) by day 14, 10/14 patients (71.4%) by day 21 and in 12/16 patients (75.0%) by day 28.

In patients receiving 200 mg FCM, the mean rise of ferritin from baseline to the peak value was 188.5 ± 67.56 μg/l (*P* < 0.001). The peak value was observed on day 2 in one patient, on day 4 in eight patients and on day 7 in three patients. A significant difference of ferritin values from baseline was observed on day 2 (91.7 ± 72.9 μg/l, *P* = 0.002), day 4 (163.0 μg/l ± 80.6 μg/l, *P* < 0.001), day 7 (128.2 ± 60 μg/l, *P* < 0.001) and day 14 (63.9 ± 78 μg/l, *P* = 0.02) (Fig. 3). 11/12 patients (92%) showed a rise in ferritin from baseline to peak of ≥100 μg/l. Ferritin levels returned to < 100 μg/l above baseline in 5/12 patients (41.7%) patients by day 7, in 8/12 patients (66.7%) by day 14, 9/12 patients (75%) by day 21 and in 11/12 patients (91.7%) by day 28.

#### TSAT

**Fig. 4:**
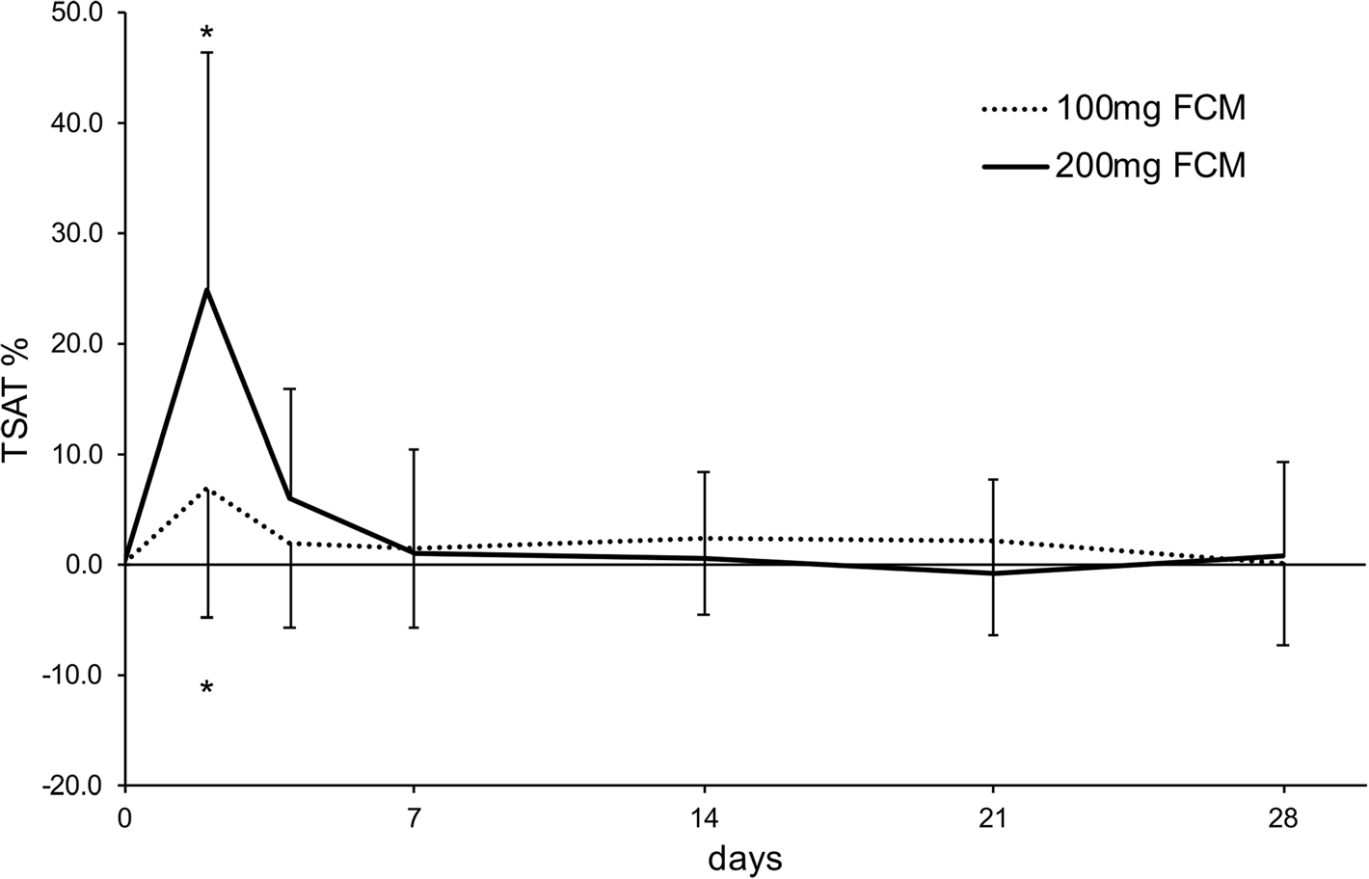
Mean change of TSAT from baseline values during 28 days after infusion of 100 mg or 200 mg FCM. Asterisks indicate a significant difference of TSAT values compared to their baseline values using a paired T-test

In patients receiving 100 mg FCM, the mean rise of TSAT from baseline to the peak value was 12.0 ± 9.7% (*P* < 0.001). The peak value was observed on day 2 in ten patients, on day 4 in two patients, on day 7 in two patients, on day 14 in one patient, on day 21 in three patients and on day 28 in one patient. A significant difference of TSAT values from baseline was observed only on day 2 (6.8 ± 11.6%, *P* = 0.01) (Fig. 4). 10/19 patients (52.63%) showed a rise in TSAT from baseline to peak of ≥10%. 9/19 patients (47.37%) showed a rise in TSAT from baseline to peak of ≥10%. TSAT levels returned to < 10% above baseline in 17/19 patients (89.5%) patients by day 7, in 12/15 patients (80.0%) by day 14, 13/15 patients (86.7%) by day 21 and in 14/15 patients (93.3%) by day 28.

In patients receiving 200 mg FCM, the mean rise of TSAT from baseline to the peak value was 23.1 ± 20.4% (*P* = 0.002). The peak value was observed on day 2 in eight patients, on day 7 in three patients and on day 28 in one patient. A significant difference of TSAT values from baseline was observed only on day 2 (25 ± 21.5%, *P* = 0.003) (Fig. 4). 10/12 patients (83.3%) showed a rise in TSAT from baseline to peak of ≥10%. TSAT levels returned to < 10% above baseline in 9/12 patients (75.0%) patients by day 7, in 9/11 patients (81.8%) by day 14, 10/11 patients (90.9%) by day 21 and in 10/11 patients (90.9%) by day 28.

#### Sensitivity analysis

The sensitivity analyses excluding either patients with missing values, or patients with a potentially relevant change of iron parameters from baseline to day 28 or both yielded essentially unchanged results (Additional file 1: Figures S3 and S4).

### Other laboratory parameters

Three patients which were included in the final analysis had a CRP level > 20 mg/l at one time point. One patient had a CRP level > 20 mg/l at 2 time points. Mean CRP values were stable throughout the study period. Hemoglobin levels were stable during the study duration and did not significantly differ from baseline at any time point. Three patients showed a rise of more than 10 g/l compared to their baseline values and three patients a decline of more than 10 g/l during the study period.

## Discussion

We found a statistically significant and potentially clinically relevant transient rise of both, serum ferritin and TSAT, after the IV administration of FCM in hemodialysis patients. As expected, the rise of both parameters was more pronounced in patients receiving the higher dose of 200 mg FCM every four weeks. Whereas roughly half of all patients receiving 100 mg FCM showed a rise in serum ferritin of > 100 μg/l, this was the case for nearly all patients receiving 200 mg FCM. While TSAT levels rapidly returned to baseline within one week in most patients, serum ferritin levels remained significantly elevated for more than two weeks in patients receiving the higher dose of FCM.

In patients with iron deficiency anemia and normal renal function, a pharmacokinetic analysis of FCM revealed a pronounced rise of serum ferritin after a single IV dose of 100, 500, 800 or 1000 mg FCM with peak values between 48 and 120 h after FCM infusion. The increase after 100 mg, however, was relatively small [16]. In these patients, baseline ferritin levels were very low with mean values between 2.1 and 5.8 μg/l in the various treatment groups. We hypothesized that dialysis patients with much higher ferritin levels at baseline might also exhibit a greater absolute increase of serum ferritin after the comparatively small FCM doses given in these patients. While several studies have evaluated the effect of repeated iron administration on subsequent rises of serum ferritin and TSAT in hemodialysis patients [18] or the correlation of maintenance iron doses with ferritin, TSAT and ESA responsiveness, data on the short-term effects of IV iron administration on these laboratory parameters are very scarce. To the best of our knowledge, only three studies have previously analyzed short-term effects of IV iron application in hemodialysis patients on ferritin and TSAT. Besarab [19] found significant elevations of both parameters until the end of the two week study duration after a single dose of 50 or 100 mg iron dextrane (*n* = 8 each). Shalansky et al. [20] evaluated iron parameters after an undefined dose of sodium ferric gluconate at a single time point at 48–72 h in 96 hemodialysis patients and at 48h and 7 days in 39 patients. They found an increase in both, TSAT and serum ferritin. While TSAT returned to baseline at day seven, ferritin remained elevated by the end of the relatively short study period. Kapoian et al. [21] serially measured serum ferritin and TSAT for 28 days in 15 patients after the administration of two doses of 510 mg ferumoxytol and found a pronounced but rapidly reversible rise of TSAT vs. a longer-lasting rise of serum ferritin with a slow decrease by the end of the study period. Thus, our study is to our knowledge the first to report serial ferritin and TSAT measurements over an entire dosing interval in hemodialysis patients on a stable iron maintenance dosing schedule.

Our data are of potential clinical relevance. Maintenance iron dosing may be preferable to bolus dosing [8, 19, 22] and has evolved to the standard of care in many dialysis centers. To maintain stable iron stores in hemodialysis patients, typical monthly IV iron doses of 100 to 400 mg are required to replace ongoing losses (through blood sampling, blood loss in the hemodialysis circuit and occult gastrointestinal losses). Although no evidence-based target levels for ferritin and TSAT exist in hemodialysis patients [10, 23], periodic sampling of ferritin and TSAT is usually performed to assess iron stores and adjust iron prescriptions. When using older IV iron preparations, such as iron dextran, relatively small single doses of iron are usually given in short intervals (e.g. weekly or even more frequently). In contrast, newer IV iron preparations such as FCM have been designed to form more stable complexes and allow for higher single doses. Thus, they are commonly given at larger doses (e.g. 100 or 200 mg of FCM) with dosing intervals between 2 weeks and one month during maintenance dosing in hemodialysis patients. Our data suggest that a minimum of 2 weeks should elapse after a 100 mg of FCM or of 3 weeks after a 200 mg dose of FCM before blood sampling for iron status evaluation. Of note, the rise in ferritin values was quite variable within a given dose group with some patients showing no increase at all, as evident from Additional file 1: Figure S1. Thus, other factors than the administered dose of iron appear to influence the magnitude of the rise in serum ferritin. Our study was too small to perform a meaningful analysis of such covariates. In clinical practice, it seems prudent to respect the above-mentioned intervals between the last iron dose and blood sampling for iron status evaluation in all patients.

Could the transient rises in ferritin represent true fluctuations of iron stores (with ongoing losses being repleted periodically) in hemodialysis patients rather than a “laboratory artifact”? Several reasons argue against this explanation. First, TSAT and ferritin exhibited considerably different kinetics with the former returning much more rapidly to baseline values. Thus, at least one of these parameters must be influenced by factors other than total body iron stores. Second, the average increases in TSAT and ferritin were considerably higher than would be expected to reflect the increase in total iron body stores after the applied FCM doses. e. g. in a study testing the safety and efficacy of FCM in dialysis patients, the administration of a mean FCM dose of 2133 mg over 11 dialysis sessions led to a rise in ferritin and TSAT by 308 μg/l and 14.2%, respectively, from baseline to four weeks after the last dose of FCM was administered [24]. Finally, even if the observed ferritin and TSAT fluctuations would partially reflect true fluctuations of iron stores, these would still need to be taken into account when evaluating iron stores and performing dose adjustments of maintenance iron treatment in hemodialysis patients.

The blood collections for the study led to a repetitive loss of small amounts of iron. Could this iatrogenic blood loss have influenced iron parameters in the study? We do not believe, because the amount of blood sampling was kept low at 10 ml per time point, amounting to 70 ml in total. This amount of blood contains 26.6 mg iron assuming a hemoglobin concentration of 110 g/l. Thus, the amount of iron lost through blood sampling for the study is relatively minor compared to the 100 or 200 mg of iron that were given. In addition, both, hemoglobin as well as ferritin concentrations and TSAT did not significantly change from baseline to week 4. Finally, if the blood draws had any effect on iron parameters, they would be expected to lower ferritin and TSAT, hence the observed increase in ferritin and TSAT would be an underestimate and the main conclusion from the study would not change. Thus, the repetitive blood sampling for the study likely had no relevant influence on the results.

Our study has several limitations. First, the number of patients analyzed was relatively small. However, a power calculation was used to estimate the number of patients required to detect clinically relevant laboratory effects. Larger patient numbers would be required for more precise estimates of the average and range of changes in ferritin and TSAT as well as potential confounding factors that affect the observed change in these parameters, but the overall conclusion would be unlikely to change. Second, although we tried to selected patients in a steady state on a stable iron dosing regimen, the iron stores of some patients might have been rising or falling from baseline to the end of the study period. This was reflected by a change in ferritin and TSAT levels from baseline to week four in some patients. However, in a sensitivity analysis excluding these patients, the results remained essentially unchanged. Third, several patients experienced signs of infection and a rise of CRP during the study period. This reflects a real world setting and was potentially aggravated by the fact that most patients participated in the study during winter months. However, whether only individual laboratory values drawn during a minor infection were excluded or patients experiencing minor infections were entirely excluded from the analysis did not affect the results, as reflected by the sensitivity analysis. Finally, since only one iron preparation was analyzed, it remains uncertain whether similar effects would be seen with other iron preparations.

## Conclusions

In conclusion, we report a significant rise of serum ferritin values after the IV administration of FCM in hemodialysis patients, which was more pronounced after a 200 mg than after a 100 mg dose. It seems advisable to temporally coordinate blood sampling for iron status evaluation with iron administration and to use “through” levels of serum ferritin to guide dose adjustments in hemodialysis patients on maintenance IV iron administration, at least in those receiving FCM.

## Additional file

Additional file 1: Supplementary Figures. **Figure S1.** Absolute change of ferritin values in relation to the respective baseline values. **Figure S2.** Relative change of ferritin values in relation to the respective baseline values. **Figure S3.** Mean change of ferritin from baseline values during 28 days after infusion of 100 mg or 200 mg FCM in the sensitivity analyses. **Figure S4.** Mean change of TSAT from baseline values during 28 days after infusion of 100 mg or 200 mg FCM in the sensitivity analyses. (DOCX 658 kb)

## Abbreviations

CKD: Chronic kidney disease
CRP: C-reactive protein
ESA: Erythropoiesis stimulating agents
FCM: Ferric carboxymaltose
HD: Hemodialysis
IV: Intravenously
RBC: Red blood cells
SD: Standard deviation
TSAT: Transferrin saturation
