# Supplementary material for: Evaluation of iron stores in hemodialysis patients on maintenance ferric Carboxymaltose dosing
Source: BMC Nephrol. 2019 Mar 1;20:76. doi: 10.1186/s12882-019-1263-8 (PMC6397449; doi:10.1186/s12882-019-1263-8)
Supplement: Supplementary file 1 — Supplementary Figures. Figure S1. Absolute change of ferritin values in relation to the respective baseline values. Figure S2. Relative change of ferritin values in relation to the respective baseline values. Figure S3. Mean change of ferritin from baseline values during 28 days after infusion of 100 mg or 200 mg FCM in the sensitivity analyses. Figure S4. Mean change of TSAT from baseline values during 28 days after infusion of 100 mg or 200 mg FCM in the sensitivity analyses. (DOCX 658 kb) [file 12882_2019_1263_MOESM1_ESM.docx]

**Additional file 1: Supplementary figures**

**Table of Contents**

**Fig S1** Absolute change of ferritin values in relation to the respective baseline values.

**Fig S2** Relative change of ferritin values in relation to the respective baseline values.

**Fig S3** Mean change of ferritin from baseline values during 28 days after infusion of 100 mg or 200 mg FCM in the sensitivity analyses.

**Fig S4** Mean change of TSAT from baseline values during 28 days after infusion of 100 mg or 200 mg FCM in the sensitivity analyses.


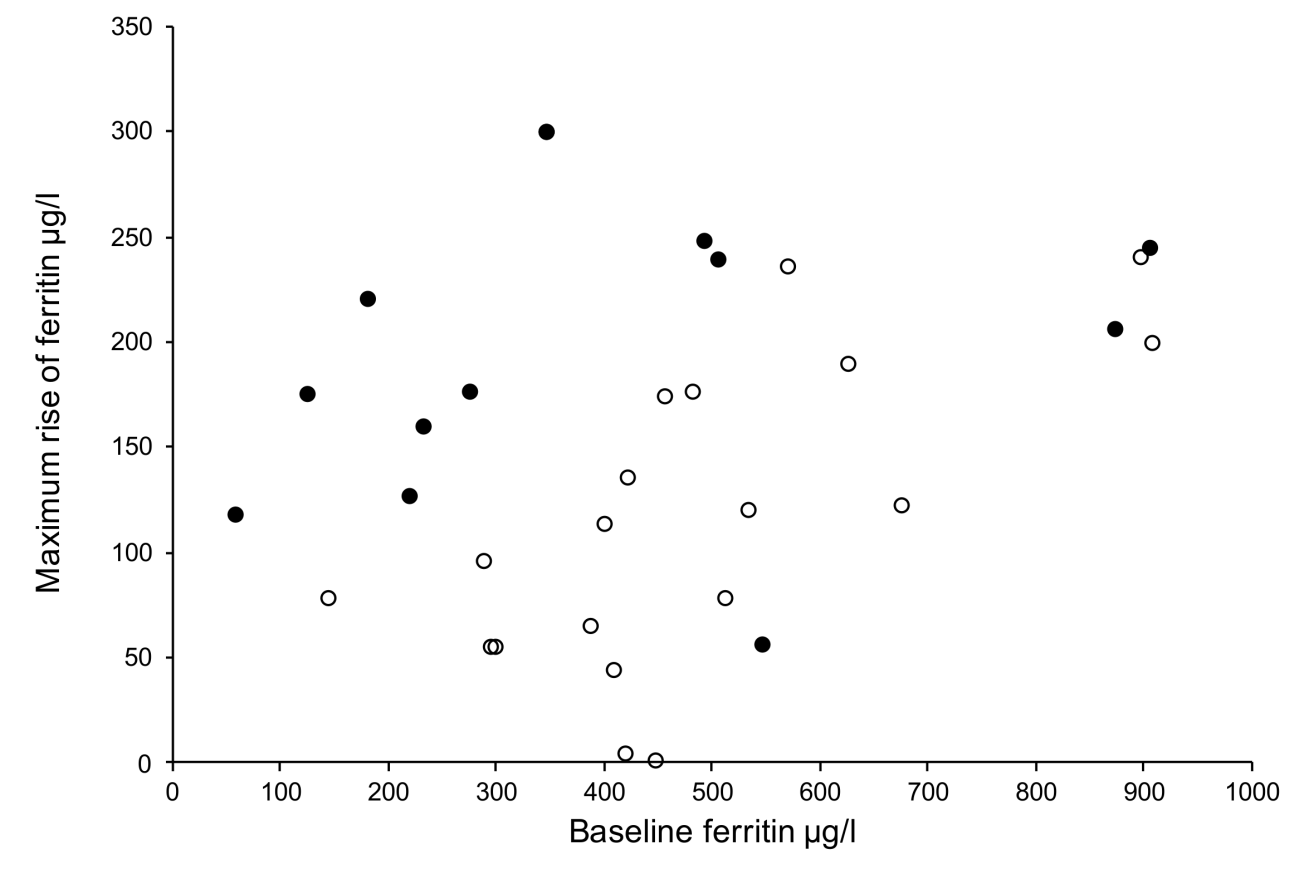


**Figure S1** Absolute change of ferritin values in relation to the respective baseline values. White dots represent patients receiving 100mg Ferinject and black dots represent patients receiving 200mg Ferinject.


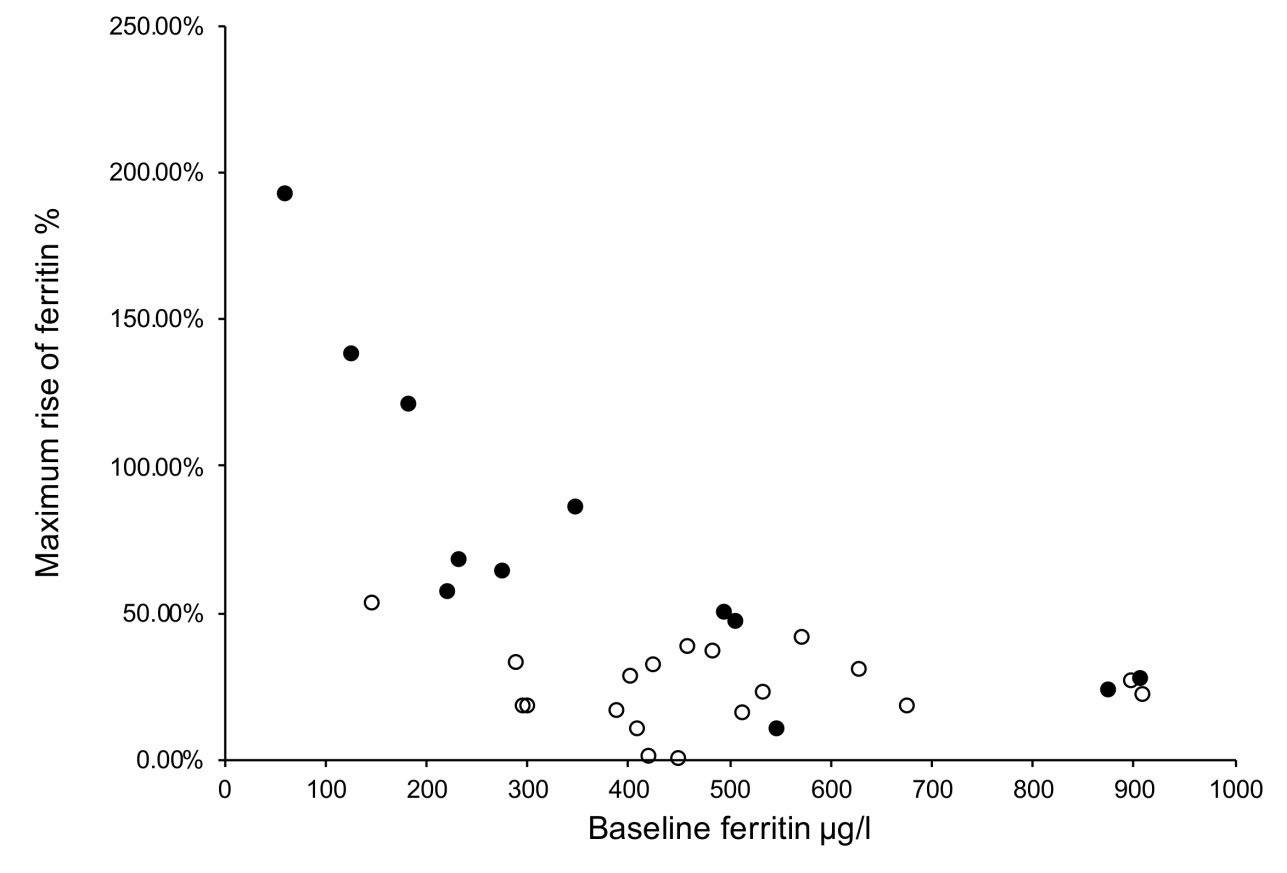

**Figure S2** Relative change of ferritin values in relation to the respective baseline values. White dots represent patients receiving 100mg Ferinject and black dots represent patients receiving 200mg Ferinject.


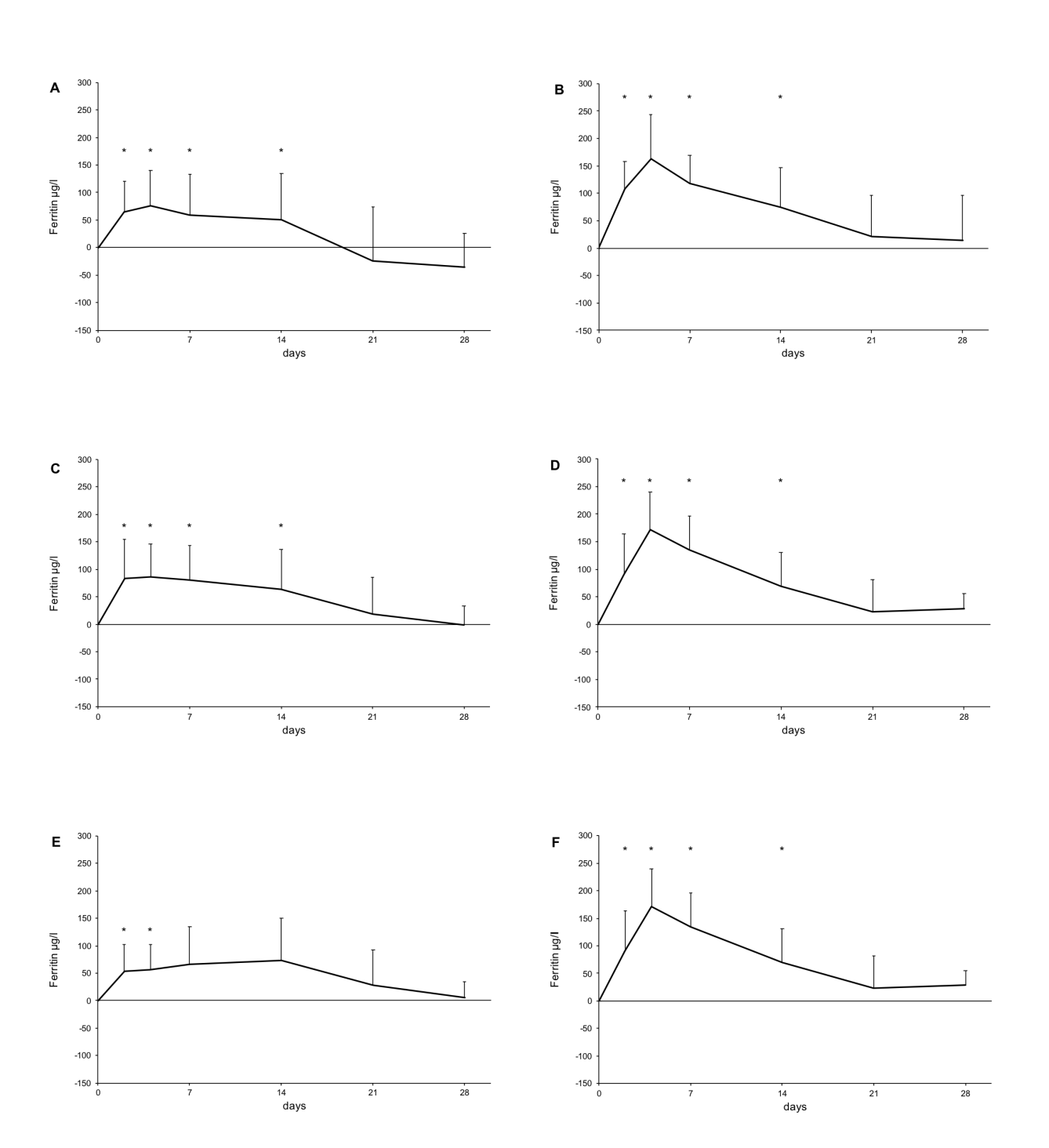


**Figure S3** Mean change of ferritin from baseline values during 28 days after infusion of 100 mg (left panels; A, C and E) or 200 mg FCM (right panels; B, D and F) in the sensitivity analyses. We excluded patients with missing values (upper panels A and B, n=11 for each study arm); patients with a relevant difference between in ferritin values (defined as a difference greater than the standard deviation of the difference from baseline to peak in all patients) from baseline to day 28 (middle panels C and D, n=11 and n=9 for the 100 mg and the 200 mg FCM study arm); or both (lower panels E and F, n=7 and n=9 for the 100 mg and the 200 mg FCM study arm). The mean changes in ferritin from baseline to the maximum value in the three sensitivity analyses were 76.5 ± 63.2 µg/l (P = 0.003), 86.5 ± 59.4 µg/l (P = 0.001) and 73.1 ± 76.8 µg/l (P = 0.058), respectively, in patients receiving 100 mg FCM and 163 ± 80.6 µg/l (P < 0.001), 171.3 ± 68.9 µg/l (P < 0.001) and 171.3 ± 68.8 µg/l (P < 0.001), respectively, in patients receiving 200 mg FCM. Asterisks indicate a significant difference of ferritin values compared to their baseline values using a paired T-test.


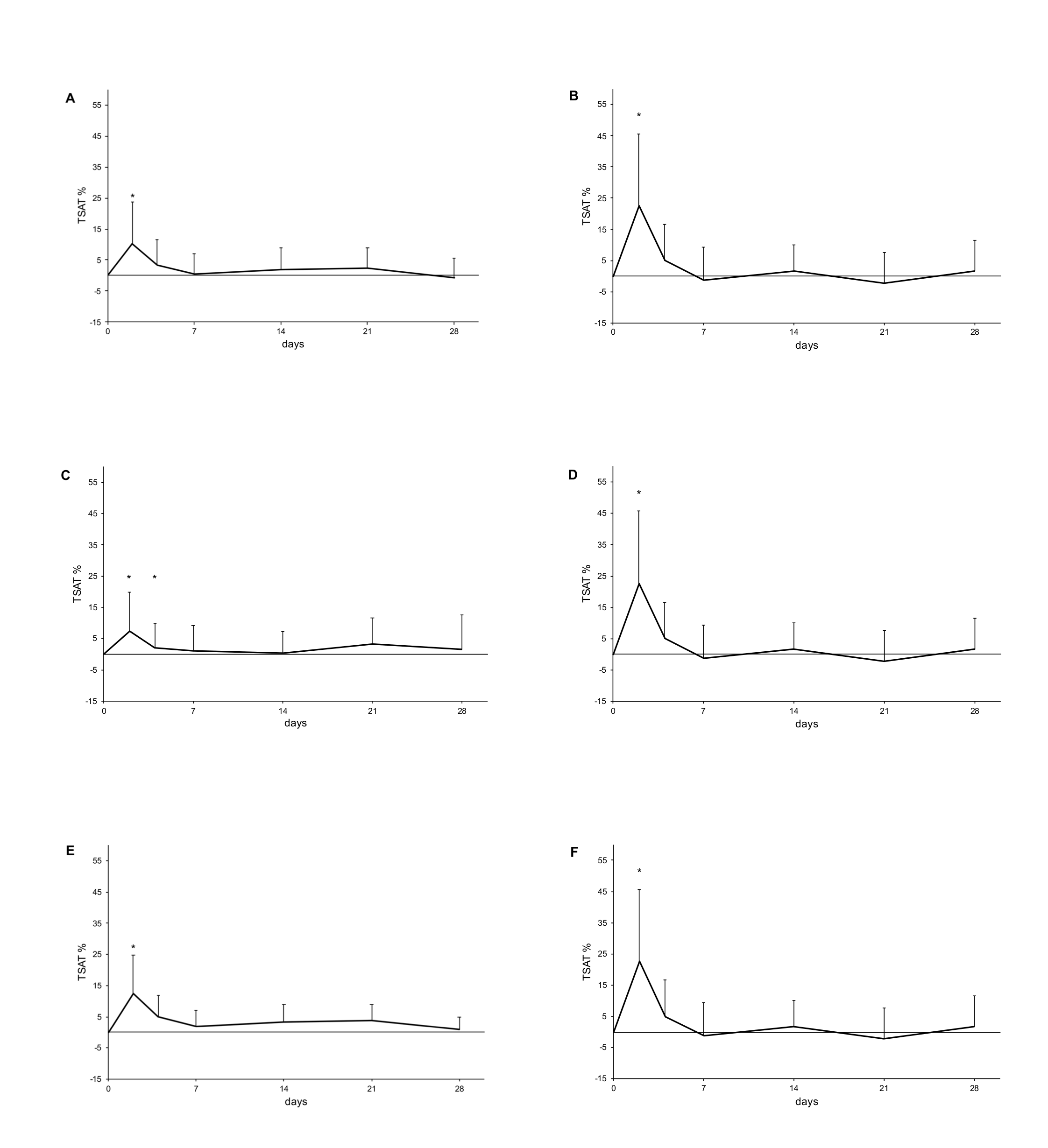
**Figure S4** Mean change of TSAT from baseline values during 28 days after infusion of 100 mg (left panels; A, C and E) or 200 mg FCM (right panels; B, D and F) in the sensitivity analyses. We excluded patients with missing values (upper panels A and B, n=10 and n=8 for the 100 mg and the 200 mg FCM study arm); patients with a relevant difference between in TSAT values (defined as a difference greater than the standard deviation of the difference from baseline to peak in all patients) from baseline to day 28 (middle panels C and D, n=15 and n=11 for the 100 mg and the 200 mg FCM study arm); or both (lower panels E and F, n=10 and n=8 for the 100 mg and the 200 mg FCM study arm). The mean changes in TSAT from baseline to the maximum value in the three sensitivity analyses were 13.8 ± 11.3% (P = 0.046), 7.3 ± 12.5% (P = 0.006) and 13.8 ± 11.3% (P = 0.046), respectively, in patients receiving 100 mg FCM and 22.6 ± 23.1% (P = 0.015), 25.2 ± 22.4% (P = 0.003) and 22.6 ± 23.1% (P = 0.015), respectively, in patients receiving 200 mg FCM. Asterisks indicate a significant difference of TSAT values compared to their baseline values using a paired T-test.
